# Preparation of Eco-Friendly Chelating Resins and Their Applications for Water Treatment

**DOI:** 10.3390/polym15102251

**Published:** 2023-05-10

**Authors:** Nicoleta Mirela Marin, Georgiana Dolete, Ludmila Motelica, Roxana Trusca, Ovidiu Cristian Oprea, Anton Ficai

**Affiliations:** 1 National Research and Development Institute for Industrial Ecology ECOIND, Street Podu Dambovitei no. 57–73, District 6, 060652 Bucharest, Romania; 2 Science and Engineering of Oxide Materials and Nanomaterials, Faculty of Chemical Engineering and Biotechnologies, University Politehnica of Bucharest, Gh. Polizu 1–7, 011061 Bucharest, Romania; 3 National Center for Micro and Nanomaterials, University Politehnica of Bucharest, 011061 Bucharest, Romania; 4 Inorganic Chemistry, Physical Chemistry and Electrochemistry, Faculty of Chemical Engineering and Biotechnologies, University Politehnica of Bucharest, Gh. Polizu 1–7, 011061 Bucharest, Romania; 5 Academy of Romanian Scientists, Ilfov Street 3, 050044 Bucharest, Romania

**Keywords:** tartrazine, amido black 10B, Amberlite IRA 402, interaction, adsorption, selectivity, metal ions, chemical recycling, circular economy

## Abstract

In the present study, two chelating resins were prepared and used for simultaneous adsorption of toxic metal ions, i.e., Cr^3+^, Mn^2+^, Fe^3+^, Co^2+^, Ni^2+^, Cu^2+^, Zn^2+^, Cd^2+^, and Pb^2+^ (M^X+^). In the first step, chelating resins were prepared starting with styrene-divinylbenzene resin, a strong basic anion exchanger Amberlite IRA 402(Cl^−^) with two chelating agents, i.e., tartrazine (TAR) and amido black 10B (AB 10B). Key parameters such as contact time, pH, initial concentration, and stability were evaluated for the obtained chelating resins (IRA 402/TAR and IRA 402/AB 10B). The obtained chelating resins show excellent stability in 2M HCl, 2M NaOH, and also in ethanol (EtOH) medium. The stability of the chelating resins decreased when the combined mixture (2M HCl:EtOH = 2:1) was added. The above-mentioned aspect was more evident for IRA 402/TAR compared to IRA 402/AB 10B. Taking into account the higher stability of the IRA 402/TAR and IRA 402/AB 10B resins, in a second step, adsorption studies were carried out on complex acid effluents polluted with M^X+^. The adsorption of M^X+^ from an acidic aqueous medium on the chelating resins was evaluated using the ICP-MS method. The following affinity series under competitive analysis for IRA 402/TAR was obtained: Fe^3+^(44 µg/g) > Ni^2+^(39.8 µg/g) > Cd^2+^(34 µg/g) > Cr^3+^(33.2 µg/g) > Pb^2+^(32.7 µg/g) > Cu^2+^ (32.5 µg/g) > Mn^2+^(31 µg/g) > Co^2+^(29 µg/g) > Zn^2+^ (27.5 µg/g). While for IRA 402/AB 10B, the following behavior was observed: Fe^3+^(58 µg/g) > Ni^2+^(43.5 µg/g) > Cd^2+^(43 µg/g) > Cu^2+^(38 µg/g) > Cr^3+^(35 µg/g) > Pb^2+^(34.5 µg/g) > Co^2+^(32.8 µg/g) > Mn^2+^(33 µg/g) > Zn^2+^(32 µg/g), consistent with the decreasing affinity of M^X+^ for chelate resin. The chelating resins were characterized using TG, FTIR, and SEM analysis. The obtained results showed that the chelating resins prepared have promising potential for wastewater treatment in the context of the circular economy approach.

## 1. Introduction

At present, industrialization is one of the main causes of pollution in the natural environment. Wastewater from chemical processes containing high concentrations of toxic metals should not be discharged into the aquatic environment without proper treatment. To solve this issue, the development of new eco-friendly materials for metal ion removal is an intensely debated issue by worldwide scientific researchers [1,2,3,4,5,6]. The efficient removal of metal ions from polluted wastewater is one of the most important steps in the de-pollution process. Consequently, the efficiency of this process depends on the selectivity of the materials used.

Conventional technologies such as chemical precipitation [7,8], membrane processes [9,10,11], ion exchange [12,13], flocculation process [14,15], activated carbon [16,17], adsorbing inorganic nanoparticles [18,19,20], etc., have been used to remove metal ions from wastewater.

Chelating resins have attracted interest over time due to their high selectivity in metal removal compared to conventional cation exchangers [21,22,23]. In addition, they offer a variety of advantages such as low operating costs, and ease of operation and they do not generate chemical sludge as do the conventional methods previously presented. Along with the advantages, the chelated resins have also numerous disadvantages. Chelated resins can quickly become saturated with pollutants. Moreover, suspended matter can block access to the porous structure of pollutants. Another disadvantage involves the regeneration costs, which can be high most of the time. They can also have similar affinity for most of the studied ions [24,25].

Therefore, the need to develop new ecological and efficient resins for the removal of heavy metal ions from wastewater will always be in the interest of technological researchers. To obtain a chelating resin, a symbiosis between the resin matrix and the functionalization agents is required [26]. Moreover, the presence of aromatic rings of the chelating agents and the resin structure based on styrene-divinylbenzene is a promising symbiosis for obtaining new chelating materials through adsorption studies [27,28,29].

The chelating mechanism involves the chelating resin and the metal ions that are retained by covalent coordinative bonds or can form hydrogen bonds with the M^X+^, but at the same time have donor atoms (sulfur, nitrogen, and oxygen) in their structure that increase the affinity for metals [24].

The latest applications of chelating resins are also presented. Gupta et al. [30] developed a new analytical method for the retention of Pb^2+^ from aqueous solutions using the chelated material obtained from the functionalization of Amberlite XAD 2 resin with gallic acid. The operational parameters were optimized and an adsorption capacity of the chelated resin for the retention of Pb^2+^ from aqueous solutions was determined to be 4554 µg/g on the tested material.

Bahsaine et al. [31] developed, for Cr^3+^ removed from polluted acid solution, a chelating ion exchanger starting from Merrifield resin that was functionalized with diethylenetriamine. The higher adsorption capacity was 38.4 mg/g and was reported under optimum experimental conditions of 300 mg/L, 120 min (25 °C).

In another study, Duan et al. [21] proposed, for Cu^2+^ and Ni^2+^ removal from wastewaters, a resin based on poly(2-acrylamide-pentanedihydroxamic acid) PAPDA that contains –CONHOH and –COOH groups. Excellent adsorption of PAPDA resin was found for Cu^2+^ and Ni^2+^. Moreover, a faster rate of adsorption was for Cu^2+^ compared with Ni^2+^.

Leonard et al. [32] have proposed, for efficient removal of Cr6+, a styrene divinylbenzene copolymer with functional group benzyl trimethyl amine. It was found 294 mg/g adsorption capacity for functionalized resin using a dosage of 1.50 g/L.

Moreover, Bringas et al. [33] have studied the adsorption/desorption of Ni^2+^ and Cu^2+^ using bis-picolyamine chelating resins from acid-stream wastewater produced by industries that contain heavy metals in high concentrations.

Elbadawyet al. [34] have investigated the adsorption behavior of Amberlite XAD-16- 1,8-(3,6-dithiaoctyl)-4-polyvinylbenzenesulfonate chelating resin for Pb^2+^ and Cd^2+^ removal. Chelating resin showed an efficiency ≈98–99% after three cycles of adsorption/desorption.

The originality of the present paper lies in preparing two chelating resins to improve the quality of acidic wastewater polluted with toxic metal ions. To the best of the authors’ knowledge, we report, for the first time, the synthesis of two chelating resins, i.e., IRA 402/TAR and IRA 402/AB 10B, for the removal of M^X+^ from complex aqueous matrices. The effect of some experimental parameters affecting the chelating process, such as pH, contact time, initial concentration of the chelating agent, and stability of the chelating resins, was studied. Thus, in the present work, a batch procedure for M^X+^ adsorption/desorption/reutilization studies on the chelating resins was applied. The resins were characterized by TG, FTIR, and SEM analysis.

## 2. Materials and Methods

### 2.1. Chemicals

**Figure 1:**
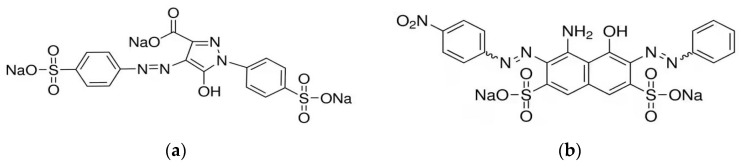
Chemical structure and IUPAC ID for TAR (**a**) and AB 10B (**b**). (**a**) 4,5-dihydro-5-oxo-1-(4-sulfophenyl)-4-[(4-sulfophenyl)azo]-1H-pyrazole-3-carboxylic acid trisodium salt (TAR). (**b**) disodium 4-amino-3-[(4-nitrophenyl) diazenyl]-5-oxo-6-(phenylhydrazinylidene) naphthalene-2,7-disulfonate (AB 10B).

The following chemicals were used for the experimental part of this manuscript: AB 10B (molecular weight (MW) 616.49 g·mol^−1^) dye content ≥80%, TAR (MW = 479.02 g·mol^−1^) dye content ≥ 85%, Amberlite IRA 402(Cl^−^), Et(OH) purity 99%, 37% HCl and 50% NaOH were acquired from Sigma Aldrich. Certified Reference Material solutions (1000 mg/L) of Mn(NO_3_)_2_, Cr(NO_3_)_3_, Cd(NO_3_)_2_, Cu(NO_3_)_2_, Ni(NO_3_)_2_, Pb(NO_3_)_2_, Mn(NO_3_)_2_, Zn(NO_3_)_2_ and Fe(NO_3_)_2_ were used applying low normality to obtain M^X+^ aqueous solutions with the following concentrations: 250, 500, 700, 1000 and 1200 µg/L, respectively. The chemical structure and IUPAC ID for TAR and AB 10 are shown in Figure 1a,b.

### 2.2. Purification and Activation of Strongly Basic Anionic Resin

Here, 6 g of dried IRA 402 resin was washed with ultrapure water in a 100 mL Berzelius flask. The washing process was repeated until the washing water was clear. The resin was kept in water to swell. When the noises produced by the expansion of the macromolecular network stopped, the resin was considered to be swollen. The resin was then quantitatively passed through a glass column—25 × 2.5 cm—height × internal diameter. The resin bed was activated with 30 mL of 4M HCl, which was allowed to flow through the column at a rate of 0.4 mL/min to activate all the functional groups of the resin, in the Cl^−^ ionic form. Finally, ultrapure water was passed through the column until a negative reaction for Cl^−^ ions was obtained in the effluent, verified with AgNO_3_. In this way, the excess of Cl^−^ ions was released from the column and only the Cl^−^ ions are found inside the resin as mobile ions (counterions) for quaternary amino groups –N^+^(CH_3_)_3_. The mean properties of Amberlite IRA 402 resin are given in Table 1 [35].

### 2.3. UV-Vis Determination of TAR and AB 10B

For the quantitative determination of TAR and AB 10B, Beer^’^s law was checked as follows: first, for TAR, the following concentrations were prepared 26, 39, 52, 65, and 79 mg TAR/L. The UV-Vis spectra against the blank sample were recorded. For the previously mentioned samples, the absorbance (A) was recorded at a fixed wavelength (λ). Thus, from the plot of A = f(concentration), the calibration curve at λ = 425 nm was determined to be A = 0.0423C – 0.0596, with R^2^ = 0.9996. For AB 10B, the spectra were recorded at λ = 618 nm and the concentrations were 20, 30, 40, 50, and 60 mg AB 10B/L. The calibration curve was determined as A = 0.0351C + 0.0524, with R^2^ = 0.9997. The LOD (limit of detection) and LOQ (limit of quantitation) were also determined from the calibration curves obtained for TAR and AB 10B, respectively. The equations used to determine the LOD and LOQ are:

(1) $$\mathrm{LOD}=3.3\frac{\sigma }{b}$$

(2) $$\mathrm{LOQ}=10\frac{\sigma }{b}$$

where: *b* is the slope of the calibration curve and σ is the standard deviation of the intercept. From the linearity data and applying the procedure described in the ICH guideline [36], the following data were obtained and are presented in Table 2.

### 2.4. ICP-MS Analysis of M^X+^

Five multielement calibration solutions of different concentrations (1–100 µg·L^−1^) were prepared by diluting a 100 mg/L multi-element calibration standard purchased from CPA Chem (CPA Ltd., Bogomilovo, Bulgaria). The calibration curves were evaluated for the following isotopes: ^52^Cr, ^55^Mn, ^56^Fe, ^59^Co, ^60^Ni, ^63^Cu, ^66^Zn, ^111^Cd, and ^208^Pb and showed good linearity over the selected concentration range, with correlation coefficients higher than 0.999. In addition, two concentrations in the calibration range (25 and 50 ppb) were checked using quality control samples (QC) prepared from a 10 mg/L multielement calibration standard 2A (p.n 8500-6940), purchased from Agilent Technologies. Both samples and calibration solutions were diluted with ultrapure water (18 MΩ·cm) obtained from a Milli-Q^®^ filtration system (Millipore, Bedford, MA, USA). For metal concentration analysis, the samples were prepared in triplicate by applying a 10-fold dilution with ultrapure water. Then, the diluted samples were immediately analyzed by Agilent 8800 Triple Quadrupole ICP-MS (Agilent Technologies, Tokyo, Japan) after a prior tuning according to manufacturer specifications. The instrument includes two mass quadrupoles separated by a helium-pressurized octupole collision/reaction cell (CRC) that removes spectral interferences. The operating conditions are given in Table 3.

### 2.5. Methodology for Evaluation Influence of Contact Time (Liquid-Solid Phases)

To evaluate the optimum contact time between the liquid and solid phase samples, dry resin samples were weighed on an analytical balance (approximately 0.25 g) into Erlenmeyer flasks. Subsequently, 0.025 L of 2000 mg/L TAR and 1000 mg/L AB 10B were added to the resin samples. The resulting mixtures were stirred for 15, 30, 45, 60, 90, and 120 min at 175 rpm (T = 25 ± 2 °C.) At the end of each contact time, the resins loaded with TAR and AB 10B were filtered and the eluent solutions were collected. The spectrometric analysis of each filtered solution was recorded and the amount of TAR and AB 10B were determined from the equations of the calibration curves.

The adsorption capacity *Q_t_* (mg/g) at time *t* was calculated using the following equation [5]:

(3) $$Q_{t}=\frac{\left(C_{i}-C_{t}\right)V\;}{m}$$

where *C_i_* and *C_t_* (mg/L) are the initial concentration of TAR and AB 10B at the time (*t*) min, *m*(g) is the mass of the dry IRA 402(Cl^−^), and *V*(L) is the volume of TAR and AB 10B. All experimental data were evaluated in duplicate and only the average data were used for *Q_t_* determination.

### 2.6. Procedure for Obtained Chelating Resin IRA 402/TAR

Them, 0.25 g of IRA 402(Cl^−^) was mixed with 0.025 L solutions of TAR with the following concentrations: 260, 390, 524, 1000, 1200, 1600, and 2000 mg/L, respectively. The batch experiments were carried out at 25 ± 2 °C, pH = 7.32, and 1 h contact time at 175 rpm mixing speed. Finally, the mixtures were filtered and the quantitative concentration of TAR was determined using the previously presented calibration curve. The amount of functionalization agent *Q_e_* (mg/g) retained in the resin mass at equilibrium was calculated using Equation (4) [37]:

(4) $$Q_{e}=\frac{\left(C_{i}-C_{e}\right)V\;}{m}$$

where *C_e_* (mg/L) is the concentration of the chelating agent’s concentration at equilibrium. All experimental data were analyzed in duplicate and only the average data were used for *Q_e_* determination.

### 2.7. Procedure for Obtained Chelating Resin IRA402/AB 10B

In order to obtain the chelate resin IRA 402/AB 10B, the following experimental steps was carried out. First, 0.25 g of IRA 402(Cl^−^) resin samples were added to the Erlenmeyer flask. Then, 0.025 L of AB 10B solution at different concentrations of 500, 600, 700, 800, 900, and 1000 mg/L were added to each Erlenmeyer flask. The mixtures were stirred at 200 rpm, pH = 7.73 for 2 h at 25 ± 2 °C. At the end of the stirring time, the samples were filtered and the concentration of AB 10B from the filtered solutions was determined by the spectrophotometric method at the λ = 618 nm.

### 2.8. Procedure for Evaluation of the Stability of Chelating Resins

To evaluate the stability of chelating resin, samples of 0.25 g chelated resin in the form of IRA 402/TAR (164 mg TAR/g IRA 402) and IRA 402/AB 10B (49.9 mg AB 10B/g IRA 402) were weighed in an Erlenmeyer flask on an analytical balance. Then, 0.025 L of 2 M HCl, 2 M NaOH and Et(OH), and 2 M HCl:EtOH = 2:1 solutions were added to the resin samples. The resulting mixtures were stirred for 30 min at 175 rpm (25 ± 2 °C). At the end of stirring, the mixtures were filtered, and the solutions were collected. The spectrum of each filtered solution was recorded and the concentration of TAR and AB 10B was determined.

(5) $$D\;\left(\%\right)=\frac{A\;}{B}\times 100$$

where: *A* is the TAR and AB 10B amount (mg) released in filtrate solution after desorption studies and *B* is the TAR and AB 10B amount (mg) that remains in the chelating resins’ mass after desorption studies.

All experimental data were collected in duplicate, and only the average data were used to determine *D* (%).

### 2.9. Procedure for Retention of Metal Ions in Chelated Resins

To study the adsorption of metal ions by the chelated resins, 0.25 g of each of the chelating resins IRA 402/TAR (164 mg TAR/g IRA 402) and IRA 402/AB 10B (49.9 mg AB 10B/g IRA 402) and 0.025 L solutions of different M^X+^ concentrations that varied between 250, 500, 700, 1000 up to 1200 µg/L were tested. The obtained mixtures were stirred for 1 h at 175 rpm (T = 25 ± 2 °C). At the end of the stirring time, each sample was filtered and the liquid phases were analyzed by ICP-MS. All experimental data were collected in duplicate, and only the average data were used to determine M^X+^ recovery *R* (%).

(6) $$R\;\left(\%\right)=\frac{\left(C_{i}-C_{e}\right)}{C_{i}}\times 100$$

### 2.10. Procedure for Recycling the Chelated Resins Exhausted with Metal Ions

The chelated resins 0.25 g of IRA 402/TAR (164 mg TAR/g IRA 402) and IRA 402/AB 10B (49.9 mg AB 10B/g IRA 402) were stirred with 250 µg/L M^X+^ (0.025 L) for 1 h at 175 rpm (T = 25 ± 2 °C). The mixtures were filtered, and solid phases loaded with metal ions (IRA 402/TAR/M^X+^ and IRA 402/AB 10B/M^X+^) were regenerated. The resulting solid phases were then stirred for 1 h with 2M HCl (0.025 L) at 175 rpm (T = 25 ± 2 °C). At the end of the stirring time, the mixtures were filtered and the M^X+^ concentrations of the filtrate solutions were determined by ICP-MS method. The solid phases obtained were kept and used in a new adsorption/regeneration experiment.

### 2.11. Solid Phases Analysis

The solid microparticles of IRA 402/TAR, IRA 402/AB 10B, IRA 402/TAR/M^X+^, and IRA 402/AB 10B/M^X+^ obtained after reaching the steady state after contact with the chelating agent and resin were dried at the laboratory temperature to remove excess of water and without altering their natural moisture content were subjected to FTIR (Nicolet iS50FT-IR (Thermo Fisher Scientific, Waltham, MA, USA), SEM (Quanta Inspect F50 electron microscope (Eindhoven, The Netherlands) and TG analysis (STA TG/DSC Netzsch Jupiter 449 F3 equipment (Selb, Germany).

## 3. Results and Discussion

The following experimental parameters were investigated to obtain IRA 402/TAR and IRA 402/AB 10B chelating resins.

### 3.1. Effect of pH of the Chelating Agent Solution

The relationship between the pH of the chelating agent solution and the adsorption capacity of the resin was studied using 1000 mg/L AB 10B and 2000 mg/L TAR. The pH of chelating agents varied between 1.9 and 10.2 by adding a few drops of HCl and NaOH (0.1 M). Thus, at pH 2.0, 5.0, and 10.0, sulfonic, carboxylic, and phenolic groups can be ionized and may be involved in interactions with the functional groups (R-N^+^(CH_3_)_3_Cl^−^) present in the resin structure. The *Q_e_* (mg/g) determined were 162.4 mg/g (pH = 2.12), 163 mg/g (pH = 4.9), 163.6 mg/g (pH = 7.3), and 162.8 mg/g (pH = 10.1) for TAR retained on IRA 402(Cl^−^). Similar results were obtained for AB 10B adsorbed on IRA 402 the flowing results were obtained: 50.5 mg/g (pH = 1.9), 49.7 mg/g (pH = 4.8), 49.9 mg/g (pH = 7.7), and 48.9 mg/g (pH = 10.2).

In conclusion, a negligible effect on the adsorption capacity was obtained by increasing the pH solution of the chelating agent on the resin masses, over a wide range of pH from ~2 to ~10. Therefore, future adsorption experiments were evaluated at pH = 7.3 for TAR and pH = 7.7 for AB 10B, representing the pH of the chelating agents dissolved in an aqueous solution. Similar results were reported in [24,38].

### 3.2. Effect of Contact Time

**Figure 2:**
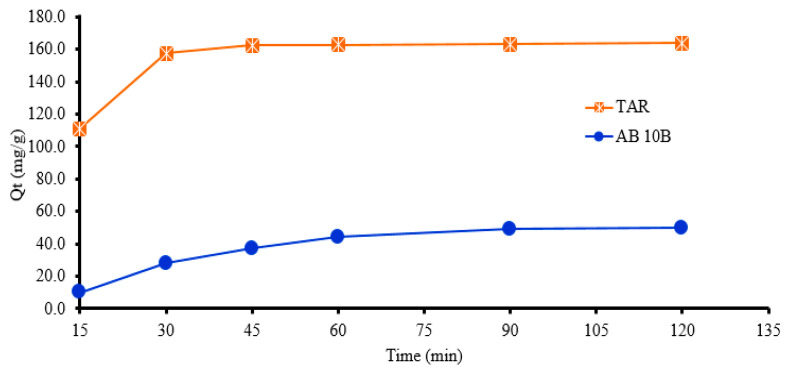
Effect of contact time on chelating agent’s removal from aqueous medium by the IRA 402(Cl^−^); The value presented represents the mean of duplicate samples with standard deviation (S*_dev_*) below 3% for all measurements presented.

To evaluate the influence of the contact time for the adsorption of TAR and AB 10B on IRA 402(Cl^−^), the following time intervals were tested: 15, 30, 45, 60, 90, and 120 min. The experimental results for the adsorption of TAR and AB 10B on the resin IRA 402(Cl^−^) as a function of the contact time are shown in Figure 2. It was observed that the saturation of the IRA 402/TAR resin is achieved in 60 min, while for the IRA 402/AB 10B resin 120 min were required. Thus, the results for obtained chelating resins showed that the saturation of the IRA 402(Cl^−^) resin with TAR is rapidly achieved. While, for complete saturation of IRA 402(Cl^−^) with AB 10B, 120 min was necessary. It was observed that the *Q_t_* values increased from 111 mg/g to 163.7 mg/g for *C_i_* = 2000 mg/L TAR and from 10 mg/g to 49.9 mg/g for *C_i_* = 1000 mg/L AB 10B. Therefore, 60 min for TAR and 120 min for AB 10B were selected as optimum contact times for future experiments to establish the equilibrium between chelating agents and the IRA 402(Cl^−^) resin.

### 3.3. Adsorption of TAR and AB 10B in Function of Effect Initial Concentration

**Figure 3:**
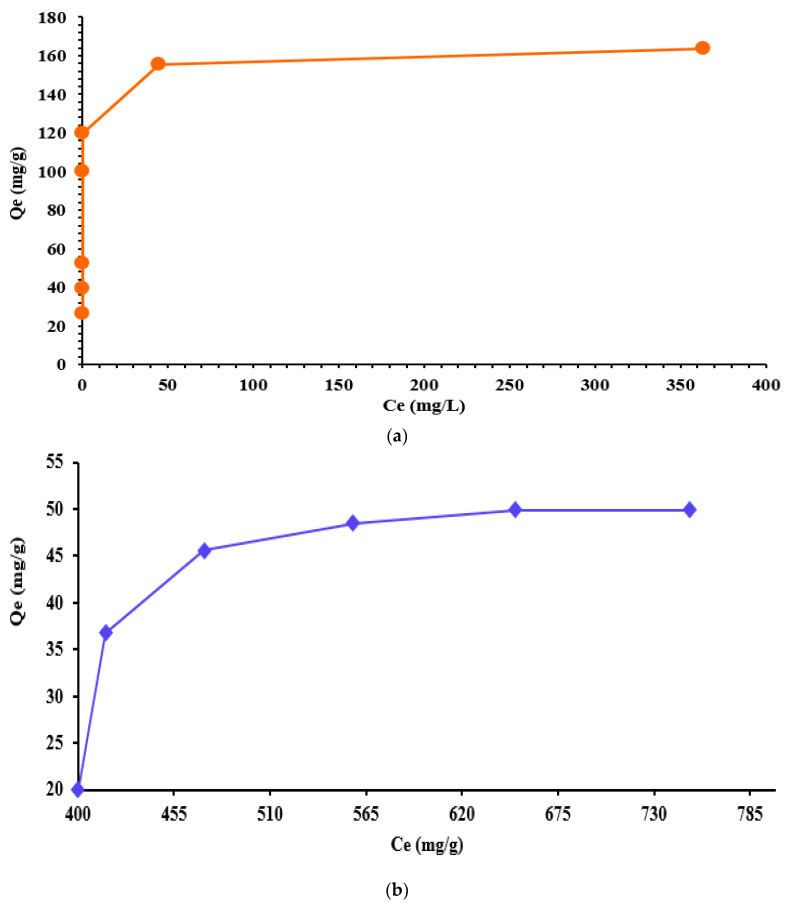
Effect of initial concentration on adsorption of TAR (**a**) and AB 10B (**b**) on the strongly basic anion exchanger IRA 402(Cl^−^). The value presented represents the mean of duplicate samples with S*_dev_* below 3% for all measurements presented.

For the obtained chelating resins, we used the strongly basic anionic resin IRA 402(Cl^−^) and chelating agents, i.e., TAR and AB 10B, which are present in aqueous solution in anionic form (ACA(SO_3_)_2_Na_2_ = ACA(SO_3_)_2_^2−^ + 2Na^+^). It is, therefore, expected that the sulfonic groups from the structure of aromatic chelating agents (ACA) will dissociate in an aqueous medium and interact with the quaternary amino groups (N^+^(CH_3_)_3_) of the Amberlite IRA 402 resin through an ion exchange equilibrium. The adsorption mechanism of chelating agents (ACA)SO_3_^2−^ on IRA 402(Cl^−^) can be described by the following Equation (7) when the interaction between sulfonic acid groups and anion exchange in Cl^−^ form (SAXCl^−^) are involved. From the experimental data presented in Figure 3a,b, it was observed that the aromatic chelating agents exhibit a high affinity towards IRA 402(Cl^−^).

2SAXCl^−^ + ACA(SO_3_)_2_^2−^ → SAX_2_(SO_3_)_2_ACA + 2Cl^−^ (7)

This reaction is irreversible, as it is necessary to use a mixture of different agents to leach chelating agents from the IRA 402 resin mass. Thus, the high stability suggests that the adsorption of chelating agents may be favored not only by an ion exchange reaction but also by π-π interactions.

It was also observed that with the increase of the initial concentration of TAR and AB 10B, the quantity of those, increased in terms of mass of IRA 402(Cl^−^). The amounts of TAR retained at equilibrium on IRA 402(Cl^−^) were to be 26, 39, 52, 100, 120, 156, and 164 mg/g for initial concentrations of 260, 390, 524, 1000, 1200, 1600, and 2000 mg/L. For AB 10B the amount retained at equilibrium was found to be 20, 36.8, 45.6, 48.5, 49.88, and 49.9 mg/g at initial concentrations of 500, 600, 700, 800, 900, and 1000 mg/L, respectively. The absorption capacity of IRA 402(Cl^−^) resin for TAR was 164 mg/g dry resin. The experimentally determined value is close to the theoretically determined resin exchange capacity [39], i.e., 200 mg/g dry resin.

Furthermore, the second resin with chelating properties was obtained using AB 10B and the same support resin IRA 402(Cl^−^). For the IRA 402/AB 10B resin, the adsorption capacity was found to be 49.9 mg AB 10B/g of dry IRA 402(Cl^−^). The previously determined value is lower than the theoretical result, which was determined to be 248 mg AB 10B/g IRA 402(Cl^−^).

This behavior can be explained as follows: AB 10B has a large chemical structure and its access to ionizable groups inside the resin can be blocked. Similar results were described at equilibrium using strongly basic Amberlite IRA 900 (type 1) and Amberlite 910 (type 2) for TAR adsorption. Moreover, adsorption behavior was studied at four interval concentrations in the range of 100–500 mg TAR/L. The maximum adsorption capacity was 49.6 mg/g for Amberlite 910 and 44.5 mg/g for Amberlite IRA 900 [40]. The effect of the initial concentration of the anionic dye AB 10B was evaluated on activated carbon with an adsorption capacity of 7.12 mg/g for initial concentrations ranging from 25 to 150 mg/L [41].

### 3.4. Evaluation of Stability for Resins in Form IRA 402/TAR and ITA 402/AB 10B

An important characteristic of chelated resins is their stability in the presence of different chemical agents. Thus, the stability of the IRA 402/TAR and IRA 402/AB 10B resins was studied in different aqueous environments: in an acidic solution (2M HCl), in a basic solution (2M NaOH), in an organic solvent medium (EtOH), and also in a combined mixture (2M HCl:EtOH = 2:1). For IRA 402/TAR and IRA 402/AB 10B, the stability was evaluated with the previously presented solutions and the spectrometric determination of the supernatant solutions was evaluated.

According to the stability studies, it was found that the IRA 402/AB 10B resin showed good stability in acidic, alkaline, and alcoholic mediums, with the desorbed percentage of AB 10B in the supernatant solutions below the quantification limit (LOQ) of the spectrometric method. Regarding the effect of the combined mixture (2M HCl:EtOH = 2:1), the stability of the IRA 402/AB 10B resin decreased, and approximately 17% of AB 10B was released in the supernatant solutions.

For IRA 402/TAR, after analysis of the supernatant solutions, the amount of TAR is not found after regeneration studies. Based on obtained data, one can say that the low concentration of Cl^−^ and OH^−^ ions from supernatant solutions of 2M (HCl, NaOH) and also EtOH medium do not affect the ion exchange equilibrium.

It can be noted that in the presence of the combined mixture (2M HCl:EtOH = 2:1), approximately 40% of the TAR retained in the resin mass was found in the supernatant solutions. When a mixture (2M HCl:EtOH = 2:1) is used, a possible explanation for the desorption of chelating agents can be that EtOH is a strong eluent that tends to solubilize chelating agents fixed on resin mass. At the same time, the resin masses are washed by the 2M HCl and chloride ions from the mixture will neutralize the amino groups produced by the synergetic effect of EtOH.

According to the results obtained, the regeneration of the chelating resins can be obtained by using the combined mixture (2M HCl:EtOH = 2:1).

### 3.5. Application of Chelating Resins

#### 3.5.1. Metal Ion Removal on IRA 402/TAR and IRA 402/AB 10B

In this study, the retention of Cr^3+^, Mn^2+^, Fe^3+^, Co^2+^, Cu^2+^, Zn^2+^, Ni^2+^, Cd^2+^, and Pb^2+^ on the chelating resins IRA 402/TAR and IRA 402/AB 10B was evaluated. Taking into account that the M^X+^ may be simultaneously present in the wastewater, their competitive adsorption was investigated. Moreover, literature studies have provided information on the fact that efficient M^X+^ adsorption is obtained when the ligand concentration inside the polymer chains is in excess of the M^X+^ present in the solution [42].

ACA was chosen for chelating IRA 402(Cl^−^) resin because can exhibit the following behavior: ACA can interact with a number of metal ions through the donor atoms existing in their molecular structure [43]. Thus, the above assumption can be taken into consideration if the –N=N–, –SO_3_Na, –OH, –NH_2_, –COONa, and C_6_H_5_OH groups do not engage in an ion exchange reaction with the studied M^X+^.

**Figure 4:**
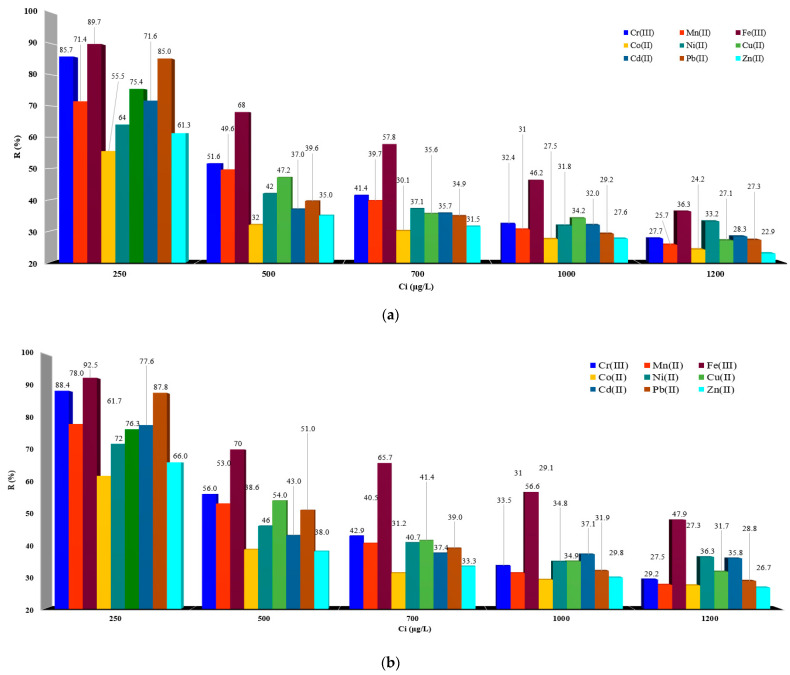
M^X+^ removed on IRA 402/TAR (**a**) and IRA 402/AB 10B (**b**) from aqueous synthetic samples; The value presented represents the mean of duplicate samples with S*_dev_* below 3% for all measurements presented.

The chelated resins IRA 402/TAR and IRA 402/AB 10B are able to remove M^X+^ due to the fact that the resin has a structure with a specific surface area and a large pore volume. Thus, the selectivity depends on the compatibility of the metal ion with the size of the pore cavity in the structure of the chelated resins. Figure 4a shows the M^X+^ retained by IRA 402/TAR resin from an aqueous medium.

Analyzing the data presented in Figure 4a, it can be seen that at 250 µg/L (initial concentration studied) for M^X+^ adsorption, Fe^3+^, and Cr^3+^ are mainly retained, but also Pb^2+^ and Cd^2+^ are well adsorbed, especially on IRA 402/TAR. At the same time, it is observed that at lower concentrations, the resin non-selectively retains all the studied metals.

So, for a solution of 500 µg/L M^X+^, IRA 402/TAR resin, Fe^3+^, and Cr^3+^ were retained in proportions greater than 50%. For the 700 µg/L solution, the IRA 402/TAR resin retains metals in proportions that vary between 31.5 and 57.8%, while for the solutions of 1000 and 1200 µg/L, the degree of M^X+^ retention decreases as a result of increasing the concentration gradient. Figure 4b shows the results obtained for M^X+^ retained in IRA 402/AB 10B resin.

As can be seen from Figure 4b, the removal of M^X+^ is obtained in very high proportions for *C_i_*), which was 250 µg/L, when more than 60% of M^X+^ studied were retained on IRA 402/AB 10B.

For the 500 µg/L solution, the retention of the metals in the IRA 402/AB 10B resin mass is observed in a greater proportion (up to 50%), excepting Co^2+^ (38.6%) and Zn^2+^ (38%) for which the retained percentages were under 50%.

For the 700 µg/L solution, the resin showed the following behavior: the metal ions studied were retained in percentages that varied between 31.2 to 43% on IRA 402/AB 10B resin. Thus, it was found that Fe^3+^ is retained in double proportion (~65%) compared to Cr^3+^, Mn^2+^, Co^2+^, Ni^2+^, Cu^2+^, Zn^2+^, Cd^2+^ and Pb^2+^, respectively.

The results obtained for the 1000 µg/L solution, the IRA 402/AB 10B has a higher affinity for Fe^3+^, which is retained in a proportion more than 56%, while for Cr^3+^, Mn^2+^, Co^2+^, Cu^2+^, Zn^2+^, Ni^2+^, Cd^2+^, and Pb^2+^ the percentages recovered varied from 29.1 to 37.1%.

For the 1200 µg/L solution, Cr^3+^, Mn^2+^, Co^2+^, Cu^2+^, Zn^2+^, Ni^2+^, Cd^2+^, and Pb^2+^ were retained in IRA 402/TAR in percentages up to 36%, while Fe^3+^ was mostly retained (~50%).

From data presented in Figure 4a,b, it can be seen that the prepared chelated resins have a significant adsorption capacity. It is noteworthy that a better adsorption capacity is observed for IRA 402/AB 10B compared to IRA 402/TAR.

#### 3.5.2. Adsorption Capacity of Metal Ions on IRA 402/TAR and IRA 402/AB 10B

**Figure 5:**
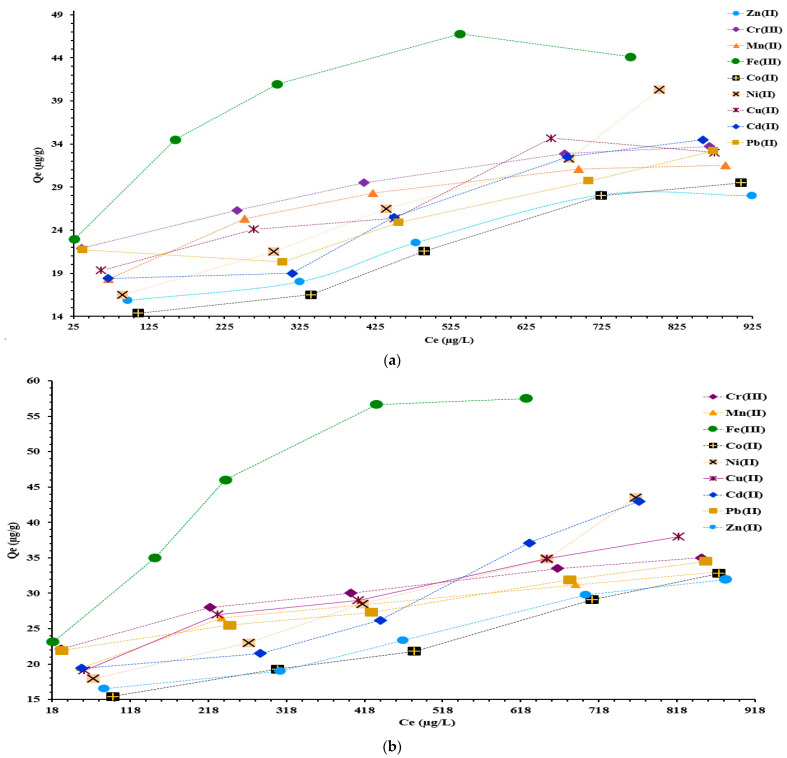
Experimental isotherm for M^X+^ removal from aqueous synthetic samples on IRA 402/TAR (**a**) and IRA 402/AB 10B (**b**); The value presented represents the mean of duplicate samples with S*_dev_* below 3% for all measurements presented.

The M^X+^ adsorption on IRA 402/TAR and IRA 402/AB 10B resins was evaluated by applying an experimental isotherm model. The adsorption capacity of metal ions on IRA 402/TAR and IRA 402/AB 10B is shown in Figure 5a,b. As can be observed, these resins have significant adsorption capacity for all metals studied one in the presence of the others. The maximum adsorption capacity of IRA 402/TAR resin for 1200 µg/L M^X+^ was more than 27.6 µg/g for all the metals studied (Figure 5a), while the IRA 402/AB 10B resin had the highest adsorption capacity for studied ions, which varied between 32.8 to 58 µg/g (Figure 5b).

However, when AB 10B is retained on IRA 402 resin resulting in IRA 402/AB 10B for M^X+^ adsorption, good behavior is obtained. This behavior may be due to the long chemical structure of AB 10B, which has a more pronounced steric effect for M^X+^ chelating compared to the TAR structure [44]. Thus, the functionalization with AB 10B led to a less porous volume in the resin structure. Moreover, M^X+^ has a good diffusion rate and ionic mobility, which is pronounced in the structure of IRA 402/AB 10B resin.

The compact TAR structure could block the access of M^X+^ into the resin structure and the adsorption capacity is affected (see Figure 5a) when comparing the results with the data presented in Figure 5b.

It is known that the binding capacity of the chelating agent for M^X+^ decreases proportionally with the ionic radius, due to the electrostatic effect. [45]. During the adsorption process, the M^X+^ must first enter the pores of the resin and then pass through the channels.

The following ionic radius are given for the studied M^x+^: Fe^3+^ (0.55Å), Cr^3+^ (0.62 Å), Co^2+^ (0.65 Å), Mn^2+^ (0.67 Å), Ni^2+^(0.69 Å), Cu^2+^(0.73 Å), Zn^2+^(0.74 Å), Cd^2+^(0.95 Å) and Pb^2+^ (1.19 Å), respectively [46]. So, the M^X+^ with the smallest ionic radius can easily be incorporated into the porous structure of the resin and interact with the functional groups of TAR and AB 10B, respectively. The results presented in Figure 5a,b suggest that the highest adsorption capacity was obtained for M^X+^ with the lowest ionic radius.

The adsorption capacity results determined under competitive conditions for the highest concentrated M^X+^ solution on IRA 402/TAR were: 44 µg Fe^3+^/g, 39.8 µg Ni^2+^/g, 34 µg Cd^2+^/g, 33.2 µg Cr^3+^/g, 32.7 µg Pb^2+^/g, 32.5 µg Cu^2+^/g, 31 µg Mn^2+^/g, 29 µg Co^2+^/g and 27.5 µg Zn^2+^/g.

Analyzing the M^X+^ adsorption results presented in Figure 5b on IRA 402/AB 10B, the following adsorption capacity values were obtained for the most concentrated solution: 57.5 µg Fe^3+^/g, 43.5 µg Ni^2+^/g, 43 µg Cd^2+^/g, 38 µg Cu^2+^/g, 35 µg Cr^3+^/g, 34.5 µg Pb^2+^/g, 32.8 µg Co^2+^/g, 33 µg Mn^2+^/g, 31.9 µg Zn^2+^/g. It was found that the IRA 402/AB 10B remove M^X+^ more with 10 µg/g for *C_i_* = 250 µg/L, with 24 µg/g for *C_i_* = 500 µg/L, with 20 µg/g for *C_i_* = 700 µg/L, with 27 µg/g for *C_i_* = 1000 µg/L and with 46 µg/g for *C_i_* = 1200 µg/L than IRA 402/TAR resin. Comparing the removal capacity for the nine cations, it can be observed that these systems have the highest affinity for Fe^3+^, regardless of the used dyes and their initial concentration. The second cation, according to the removal rate is often Cr^3+^, at least at low concentration (*C_i_* = 250, 500, or 700 µg/L), but at higher *C_i_* (1000 and 1200 µg/L) the second most removed cations are Ni^2+^, Cu^2+^, Cd ^2+^, or Pb^2+^, which means that the removal is based on a complex mechanism involving thermodynamic or kinetic control.

Therefore, both chelated resins have significant adsorption capacity in mixed solution for all the M^X+^ studied and may be promising materials in the wastewater treatment process.

Applications of chelating resins for M^X+^ removal were also presented in other studies. Li et al. [45] prepared a chelate material for Pb^2+^ extraction from aqueous medium. The adsorption capacity was 21.24 mg/g at an initial concentration of 50 mg/L [45]. The adsorption behavior for Pb^2+^ and Cd^2+^ removal on m-phenylenediamine-modified Amberlite XAD-4 resin was tested. To obtain chelating resin, Amberlite XAD-4 was purified with 4M HCl and subsequently modified with m-Phenylenediamine, a chelate agent that forms complexes with Pb^2+^ and Cd^2+^ on the resin surface. The metal concentrations studied ranged from 5 to 100 mg/L. It was found that for both metals studied, the removal efficiency was 99.50% for Pb^2+^ and 99.35% for Cd^2+^ [47].

In conclusion, the metal concentrations evaluated on both chelated resins tested in our paper are frequently found in polluted effluents and sometimes difficult to treat by conventional methods. The management of this type of effluent can be resolved using chelating macroreticular resins, which have many advantages together with simple operation and good regeneration reported to conventional methods [48].

### 3.6. Reusability of Chelating Resins

The acidic effluents polluted with metal ions can be treated by applying the principles of the circular economy. This involves reducing the metal concentration and recovering the materials for subsequent reuse [49].

**Figure 6:**
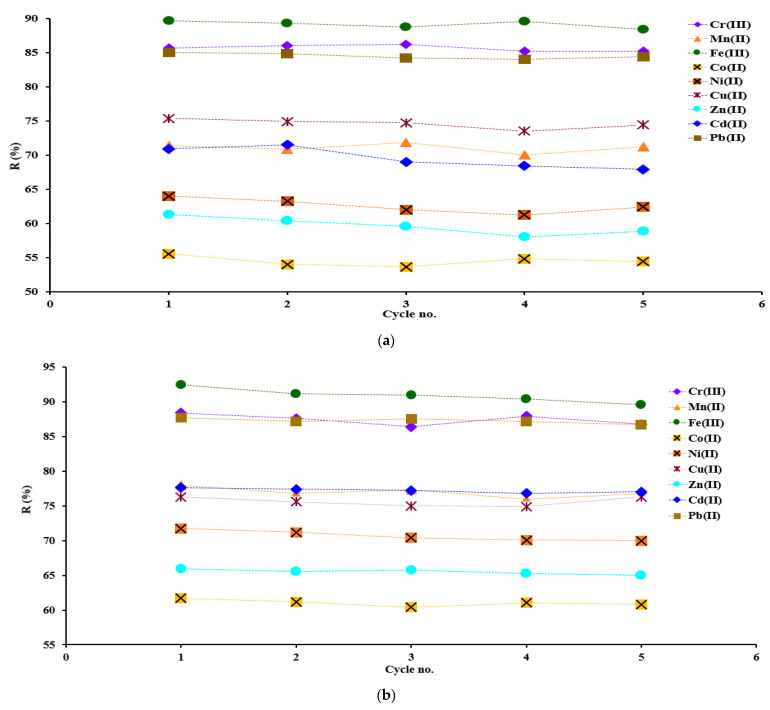
Reusability of IRA 402/TAR (**a**) and IRA 402/AB 10B (**b**) for M^X+^ adsorption; The value presented represents the mean of duplicate samples with S*_dev_* below 3% for all measurements presented.

The chelated resins loaded with metal ions were regenerated and reused in five adsorption/desorption cycles. It was found that after a new use from one to five, the adsorption of metals is maintained around the same percentage range. From the data presented in Figure 6a,b, it is observed that the adsorption capacity of the chelated resins for the metal ions studied remains constant (variation maximum being up to 2%) after 5 adsorption/desorption cycles, results that suggest the feasibility of the resins used.

Similar studies on the reusability of chelating resins were carried out when Amberlite XAD-2 loaded with o-aminophenol was subjected to up to 16 cycles for adsorption/desorption of Cu^2+^, Cd^2+^, Co^2+^, Ni^2+^, Zn^2+^, and Pb^2+^. A good feasibility of the resin used for multiple reuses was observed [50]. In another study, the recyclability of aminophosforic chelating resin for vanadium adsorption/desorption was tested. The performance of the recyclability of the resin was studied for nine cycles of vanadium adsorption/desorption [51].

### 3.7. Solid Phases Analysis

#### 3.7.1. TG Analysis

**Figure 7:**
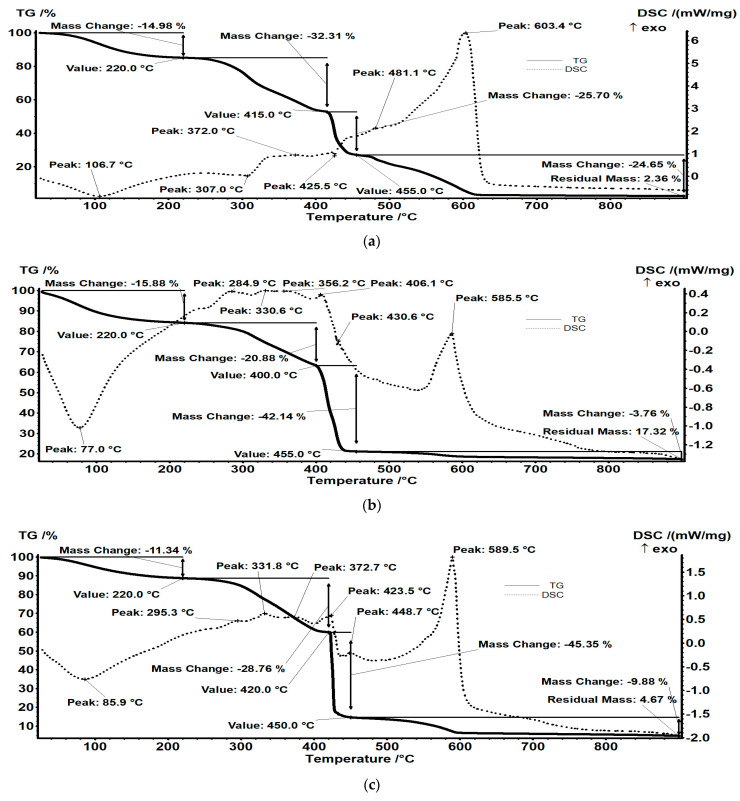

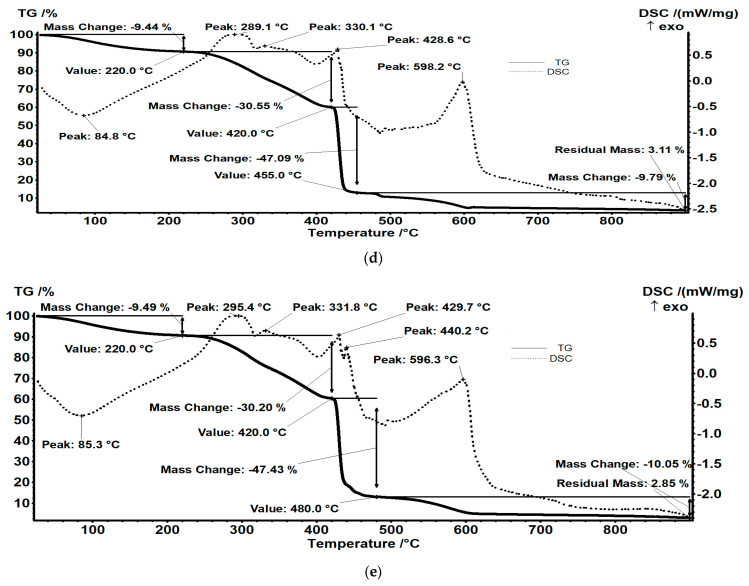
Thermal degradation of IRA 402(Cl^−^) (**a**), IRA 402/TAR (**b**), IRA 402/AB 10B (**c**), IRA 402/TAR/M^X+^ (**d**), and IRA 402/AB 10B/M^X+^ (**e**).

The resin samples show a multiple-step degradation pattern, which can be simplified to about four intervals. The degradation steps of the solid resin samples, i.e., IRA 402(Cl^−^), IRA 402/TAR and IRA 402/AB 10B, IRA 402/TAR/M^X+^, and IRA 402/AB 10B/M^X+^ are shown in Figure 7a–e. The percentage mass losses are shown in Table 4. The thermal analysis TG-DSC for the samples was evaluated. The samples were placed in an open alumina crucible and heated from room temperature up to 900 °C at 10 K·min^−1^, under a flow of 50 mL min^−1^ in dry air. An empty alumina crucible was used as a reference.

Up to RT-220 °C, the resin microparticles undergo a dehydration process, the recorded mass loss being 14.98%. This is assigned to the elimination of the physically bound water molecules from the interior of the microparticles [52]. The process is accompanied by an endothermic process, with a minimum on the DSC curve at 106.7 °C.

Between 220–415 °C the organic part undergoes a series of degradation processes (with a recorded mass loss of 32.31%) breaking of the main polymer chain, fragmentation to smaller molecules, and oxidation of the fragments. The resulting effect is, therefore, a combination of individual reactions, with a predominance of exothermic reactions.

The residue decomposes violently, between 415–455 °C, with a loss of mass of 25.70%, the process probably consisting of decomposition and oxidation of fragments as suggested by the DSC curve. The carbonaceous mass burns after 455 °C as indicated by the strong, asymmetric exothermic effect occurring at 603.4 °C.

#### 3.7.2. FTIR Analysis

**Figure 8:**
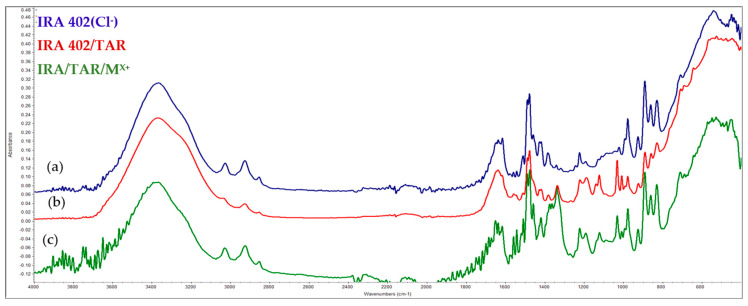
FTIR spectra of: (**a**) IRA 402(Cl^−^), (**b**) IRA 402/TAR and (**c**) IRA/TAR/M^X+^.

**Figure 9:**
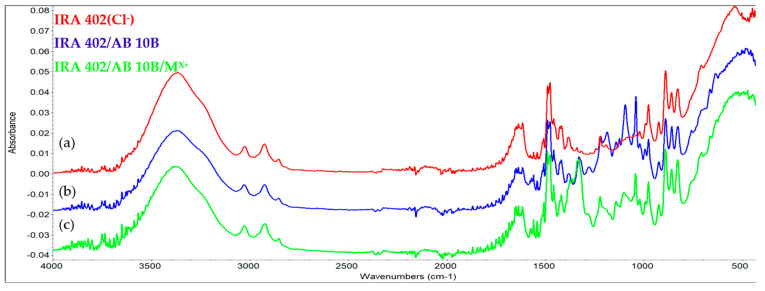
FTIR spectra of: (**a**) IRA 402(Cl^−^), (**b**) IRA 402/AB 10B and (**c**) IRA 402/AB 10B/M^X+^.

Infrared spectroscopy was also used to evaluate the changes that occurred as a consequence of the absorption of the dyes and metals. As a first conclusion, it can be seen that significant changes occur as the gradual absorption of the dye and metals occurs onto the resins. Figure 8 and Figure 9 highlight the specific spectra of the reference resin (IRA-402), IRA-402/dye complex, and IRA-402/dye/M^X+^.

**Figure 10:**
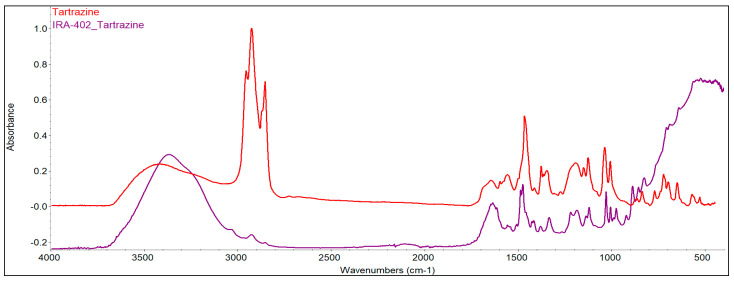
FTIR spectra overlapping of TAR (red) and IRA 402/TAR (magenta).

**Figure 11:**
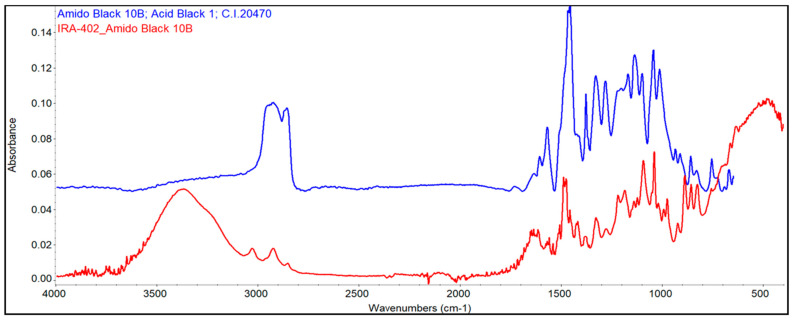
FTIR spectra overlapping of AB 10B (blue) and IRA 402/AB10B (red).

It is important to mention that TAR adsorption leads to a significant change in the shape of several spectral regions (especially the region 1100–1250 cm^−1^ specific to the sulfur-oxygen vibrations or the significant decrease of the peak at 1557 cm^−1^ –N=N– vibration) as well as the shifts of the specific bands of the tartrazine (such as the shift of the peak from 1467 to 1475; 1334 to 1350 cm^−1^) due to the strong interactions that occur between the resin and the TAR (Figure 10). This is also supported by the reusability data, which shows that five successive cycles slightly change the absorption capacity of the metal ions (<2%). Similar results can be observed for the second dye, called AB 10B (Figure 11).

When cations are additionally adsorbed, the specific bands of the complexing dyes are changed, indicating that the retention of the cations is mainly happening due to the dyes and not due to interaction with the resin. For instance, in the case of the IRA-402/AB 10B, the most visible change is in the region 1250–1400 cm^−1^, or the peaks centered at 1099, 1190 cm^−1^, whereas in the case of IRA-402/TAR, due to the retention of the metals, the region 1282–1405 cm^−1^ is strongly affected (along with the peaks from 980, 1034, 1224 cm^−1^, etc.).

#### 3.7.3. SEM Analysis

**Figure 12:**
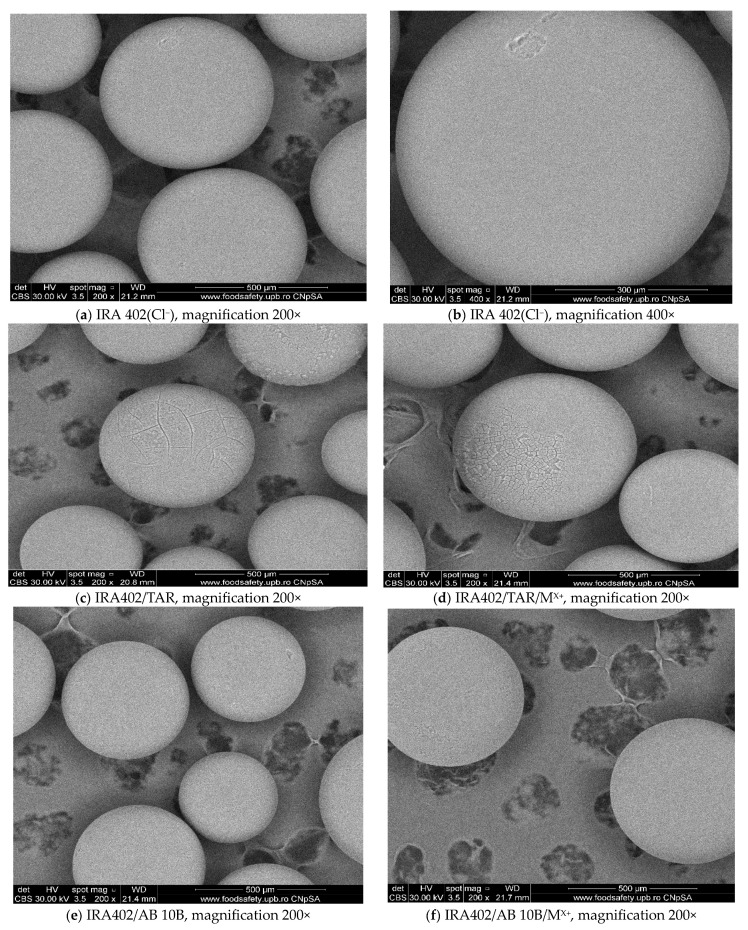
SEM image of IRA 402(Cl^−^) (**a**,**b**), IRA 402/TAR (**c**), IRA 402/TAR/M^X+^ (**d**), IRA402/AB 10B (**e**) and IRA402/AB 10B/M^X+^ (**f**).

Based on the SEM images it can be concluded that the resin is microparticulate, with a diameter of 300–500 µm and a good spherical shape. No mechanical deformation or other changes regarding resin microparticle stability are observed during the chelating process and subsequently in M^X+^ removal (see Figure 12a–f).

In addition, SEM images obtained for IRA 402(Cl^−^) after functionalization with TAR and AB 10B provide a uniform microporous structure on the regular spheres (Figure 12c,e). After M^X+^ removal, small differences are highlighted in terms of the microporous structure when the surface becomes rougher (Figure 12d,f). This observation is more evident for IRA 402/AB 10B/M^X+^ resin when compared to IRA 402/TAR/M^X+^ (Figure 12d,f).

## 4. Conclusions

The paper aimed to obtain new chelated resins for the adsorption of toxic metal ions from aqueous environments. The chemical modification of the styrene-divinylbenzene copolymer leads to the desired functionalized resin. The microporous resin ensures good retention of aromatic chelating agents due to its structure based on styrene divinylbenzene as well as the ion exchange reaction that occurs between the ion exchange resin (R-N^+^(CH_3_)_3_Cl^−^) and the anions from aqueous medium represented by aromatic chelating agents. It was found that slightly different contact times are required for chelating IRA 402(Cl^−^) resin with TAR and AB 10B. Thus, the optimum time for obtaining the chelated resin IRA 402/TAR was 60 min, while for obtaining IRA 402/TAR, a slightly longer contact time is required (120 min).

The experimental study showed that these systems are quite stable in alkaline and acidic aqueous solutions, whereas the mixture (2M HCl:EtOH = 2:1) has a degrading effect and the chelating agents are slightly removed from the synthetic (~17%).

Chelation interactions are likely to be the mechanism involved in M^X+^ adsorption together with the porosity of chelated materials studied. The concentrations of metal ions studied to test the chelated resins are frequently found in real water samples. The adsorption capacity *Q_e_*, (µg/g) of M^X+^ on the IRA 402/TAR and IRA 402/AB 10B were calculated for concentrations that were varied in the range of 250 to 1200 µg/L. Furthermore, the effect of concentration significantly influenced the amount of M^X+^ retained in the mass of the chelating resins prepared. Our study focused on the evaluation of both chelating resins, although the IRA 402/AB 10B resin showed the best adsorption capacity for the M^X+^ studied compared to IRA 402/TAR. The resin in IRA 402/TAR and IRA 402/AB 10B forms proved to be efficient materials for the removal of metal ions from an aqueous acidic medium.

Moreover, the IRA 402/TAR and IRA 402/AB 10B showed excellent reusability, suggesting that the prepared chelating resins can be employed for M^X+^ removal from complex effluents.

## Figures and Tables

**Table 1:** Characteristic data for Amberlite IRA 402 resin.

Type	Strongly Basic Anion Exchanger
Matrix	Styrene-divinylbenzene copolymer
Ionic form as shipped	Chloride (Cl^−^)
Physical form	Yellow Translucent beads
Total ion exchange capacity	≥1.2 eq/dm^3^
pH range	0–14
Moisture holding capacity	49–60% Chloride form
Harmonic mean size	0.6 mm–0.75 mm; (20–25 mesh)
Maximum operating temperature	77 °C

**Table 2:** LOD and LOQ of TAR and AB 10B of the UV-Vis method.

Parameter	TAR	AB 10B
LOD (mg/L)	0.07	0.10
LOQ (mg/L)	0.21	0.31

**Table 3:** ICP-MS operating conditions.

Parameter	Operating Condition
RF power	1150 W
Sample Depth	8.0 mm
Carrier gas	1.00 L/min
Nebulizer pump	0.10 rps
Spray-Chamber Temperature	2 °C
Cell gas flow (He)	7 mL/min
Dwell time	10 ms (Cr, Fe) and 12 ms (Mn, Co, Ni, Cu, Zn, Cd, Pb)
Sampling cone	Ni-tipped with Cu base
Skimmer cone	Ni

**Table 4:** Thermal mass loss of IRA 402(Cl^−^) and chelating resin before and after adsorption of M^X^.

Sample	Water Content (Mass Loss RT-220 °C)	Mass Loss 220–415 (420) °C, %	Mass Loss 415–455 °C, %	Mass Loss 455–900 °C, %	Residual, %
IRA 402(Cl^−^)	14.98	32.31	25.70	24.65	2.36
IRA402/TAR	15.88	20.88	42.14	3.76	17.32
IRA402/AB10B	11.34	28.76	45.35	9.88	4.67
IRA402/Tar/M^X+^	9.44	30.55	47.09	9.79	3.11
IRA402/AB10B/M^X+^	9.49	30.20	47.43	10.05	2.85

## Data Availability

Data are available on request from the authors.
